# Modern Sociology

**Published:** 1899-12-09

**Authors:** 


### Modern Sociology.

### THE RENEWAL OF LYNCHING.

During the last few years the civilised world has been horrified by frequent accounts of the lynching of negroes in the Southern States of America. The rough justice which goes by the name of lynching—the execution of criminals without formal trial and condemnation—is often a necessary stage in the progress of a community. Where rough characters abound and the executive is weak, as often happens in newly-settled districts, it may be excusable for the community as a whole to execute judgment sharply and speedily. But the practice is a dangerous one, leading to frequent miscarriages of justice, and, moreover, tending to make the whole community take a delight in bloodshed, which soon reduces them to the level of savages.

It is this demoralisation of the entire population that threatens to result from the recrudescence of lynching. The crime for which the negroes are said to suffer is one that excuses condign punishment. We are always assured that lynching never takes place except in a case of rape committed by a black man on a white woman. Now, one can easily understand the indignation of the outraged woman's friends, and also her own reluctance to come into a public court of justice and there narrate all the details of the outrage. It is this that has so long made people, otherwise just and merciful, refrain from condemning lynching as it deserves. But the result has been a reckless shedding of blood, often for very doubtful cause, and a growing tendency to murder negroes on the slightest provocation, or none. The now historic lynching of Sam Hose is an example of this. Sam Hose, a negro, had killed a white farmer, and, it was said, outraged his wife. The murder was admitted, but the man denied the other crime. But, regardless of his protests, a crowd of infuriated whites chained him to a tree, mutilated him in a ghastly manner, and then burned him alive. While he was being fastened to the tree, the poor wretch, doubtless in the hope that he might escape, said that he had been bribed to murder his victim by a negro preacher named Strickland. The result of this statement was, not that Hose escaped, but that when his agony was over the crowd went to Strickland's house and murdered him. There was no examination into the truth of the statement. Strickland was accused; he was black, he was killed. Other cases might be quoted, a little less brutal in detail, perhaps, but as little founded on reason or proof of guilt.

And one cannot but ask these avengers of outraged women what lessons they have taught the black race that dwells among them with regard to women's honour. The existence of mulattos, quadroons, octoroons, down to those white-skinned men and women, in whom only experts on the subject can see any trace of African blood, might be held to be answer enough. The admixture can in many cases be traced back to the days of slavery. Even so, the fact is sufficient to create a bitter feeling in those who, with the blood of the ruling race in their veins, are doomed forever to the status of the despised one. But are sexual relations between white men and coloured women unknown to day? And where they exist, can it always be assumed that the women have consented to them without violence or fear? Is there a case on record where the whites of a community have risen in wrath and lynched one of their number because he had violated a coloured woman? If lynching is rough justice, let it be equal justice, and not reserved for one race only. But such records as we have show the opposite. One case may be quoted. A young white man, the son of a judge, seduced the wife of a coloured man named Julian. Julian having found out the fact, uttered some threats of vengeance. Fearing the consequences, the judge had the negro arrested on some excuse. Brought into court, Julian drew a pistol and shot the judge, and then, in the ensuing confusion, managed to escape. The infuriated crowd in the court house rushed out, and lynched, not the culprit, who could not be found, but his three brothers, who were at work in a field and knew nothing of what had taken place. They then went on to the house where Julian's mother was employed as a servant, and would have killed her if her employer had not protected her. A community that has given itself up to such meaningless lust of blood as this case shows, is certainly not to be trusted to execute justice without the intervention of the ordinary courts of law. Admitting the negro's crime, the situation is not mended by the killing of three innocent men; while on Julian's side it may be pleaded that he had suffered a wrong for which he would receive no redress, and that he was to be imprisoned in order that the seducer might go his way without cause for fear. Nor would it tend to make him more patient under his wrongs to reflect that if he had been the aggressor, and had violated the sanctity of a white man's hearth, he would have been killed with as little delay as was compatible with adding some fiendish torture to death. When the white men break the law they can scarcely expect the black to be patient and law-abiding.

And herein lies the chief danger. Judge Lynch always gives effect to the verdict of the majority. The whites have called him in, but who shall say that the blacks will not do the same? And if they ever follow on a large scale the lawlessness of their betters we shall see such a war as could be paralleled only by the worst excesses of the Indian mutiny.

Moreover, while southern people assure the world that rape is the only crime to punish which lynching is resorted to, outsiders in the south tell a very different story. They say that negroes are lynched for petty thefts, and even on mere suspicion of crime. At the bottom of it all there is race hatred. The mischief-makers are the "poor whites." These think that, in spite of emancipation, the negroes should be still what they were in the days of slavery. They would have them work, but not for themselves. Now, many negroes are lazy, but there are among them some who have attained some degree of wealth; while others are educated, and claim the status of educated men. Now, this the "poor whites," who also are lazy, and too proud to work, grudge them. They quite sincerely think they have a grievance against this coloured "inferior" race for not being content with its inferiority, for claiming the rights of citizenship, for cherishing ambitions, and achieving the object of them. This is at the root of the matter, and the lynchings are intended to warn the negro to "keep in his proper place." There is no doubt the situation is a difficult one. We Britons know how hard it is for two races of different extraction, temperament, and degree of civilisation to dwell together in amity. But we have learned by bitter experience that it does not make for peace to allow one race to override justice, and give rein to its lowest passions. May the whites of the Southern States also learn that lesson before intolerable wrong arouses her negro population to take the vengeance which their numbers makes possible!

